# Modular Software Architecture for Local Smart Building Servers

**DOI:** 10.3390/s21175810

**Published:** 2021-08-29

**Authors:** Lamine Lagsaiar, Isam Shahrour, Ammar Aljer, Aziz Soulhi

**Affiliations:** 1 Civil and Geo-Environmental Engineering Laboratory, Lille University, 42 Rue Paul Duez, 59000 Lille, France; isam.shahrour@univ-lille.fr (I.S.); ammar.aljer@univ-lille.fr (A.A.); 2 Laboratory of System Analysis, Information Processing and Industrial Management (LASTIMI), Mohammed V University, Rabat 10000, Morocco; soulhi@enim.ac.ma

**Keywords:** smart building, software architecture, IoT, hardware, raspberry, nodejs

## Abstract

This paper presented the architecture and construction of a novel smart building system that could monitor and control buildings’ use in a safe and optimal way. The system operates on a Raspberry local server, which could be connected via the cloud technology to a central platform. The local system includes nine modules that inter-communicate. The system detects sensor faults, and provides a friendly interface to occupants. The paper presented the software architecture IoT used for the building monitoring and the use of this system for the management of fifteen social housing units during a year. The system allowed the investigation of indoor comfort and both energy and hot water consumptions. Data analysis resulted in the detection of abnormal energy consumptions. The system could be easily used in buildings’ management. It works in a plug-and-play mode.

## 1. Introduction

Research into smart buildings has increased dramatically over the past decade. Several architectures and technologies have been tested and implemented to show the importance and promising functionalities of smart buildings, especially with the arrival of the Internet of Things, which enters into the daily functioning of many industries [1], and it presents itself with a set of new applications and technologies.

Scholars tackled different topics related to the conception of a smart building monitoring system. Alexakis et al. [2] used natural language processing to control and monitor sensors. They relied on integrating several third-party APIs such as the Dialogflow API and open-source technologies that aim to develop new and very fast smart building solutions. They chose Wemos D1 Mini V3 as microcontrollers with integrated Wi-Fi chips onboard. Lin et al. used Nodemcu as microcontrollers and Raspberry Pi as a Gateway [3]. Balikhina et al. used the Intel Edison development platform as a gateway that uses MQTT as a protocol of communication [4], similar to that proposed by Alexaki et al. [2].

Rihab al. [5] investigated IoT security and privacy in smart buildings. They presented an unprecedented contribution by offering a login module to manage user registration and login operations more securely using a multi-factor authentication method based on a password, liveliness detection, and facial recognition. Chen et al. [6] proposed architecture for an intelligent building control system to improve the energy efficiency of buildings with a proof-of-concept implementation. Dutta et al. [7] presented a solution based on fog and cloud architecture that uses open-source software to reduce costs without compromising the system’s quality of service. On the other hand, Al-Ali et al. [8] proposed a smart home system based on IoT-Big data to manage energy consumption using remote monitoring and control through a mobile application. Bellagente et al. [9], Ock et al. [10], and Uribe et al. [11] proposed solutions for energy management, but they did not present the software architecture of these systems. Few researchers tackled smart building gateways. Yan et al. [12] compared recent smart gateways used in smart buildings. The comparison was based on the operating systems, wireless communication protocols, and security. 

The literature misses papers addressing the architecture of the internal local server, their components, and their interaction. This research aimed at filling this gap. It presented a comprehensive smart building system, including the software architecture. The performances of this system were investigated through monitoring fifteen social housing apartments for one year. 

The paper is organized into five sections. Section 1 introduces the research problem and the related works. Section 2 proposes the smart building architecture and materials. Section 3 presents the system software architecture. Section 4 presents the case study. Finally, Section 5 offers the major outcome of this research and its perspectives.

## 2. Smart Building Architecture & Materials

This paper focused on the use of the smart building to provide services to both occupants and managers. These services are related to monitoring (i) the indoor comfort including temperature, humidity, lighting, noise, and air quality, (ii) water and energy consumption, and (iii) occupants’ behavior. Analysis of collected data aimed at ensuring comfortable conditions to occupants, improving their safety, and reducing energy and water consumption.

Table 1 presents a comparison between the proposed system and the smart building systems in the literature, in terms of hardware, software specifications, and proposed services.

**Figure 1:**
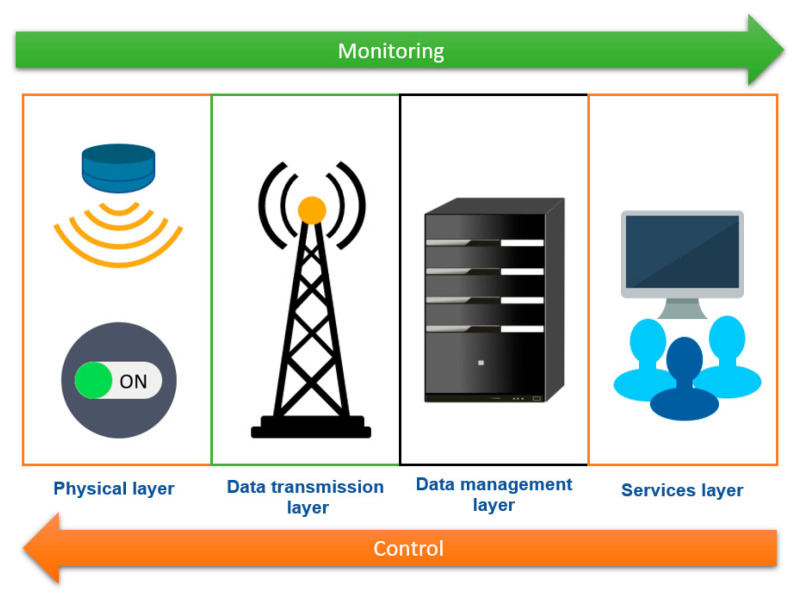
The architecture of the smart building system.

The architecture of the smart building was based on four layers, as shown in Figure 1. All the layers are connected to change data and queries. We distinguish two types of data flow:

- The Monitoring data: allows monitoring the indoor parameters (comfort, consumption, security) of the buildings with the help of sensors (Physical Layer) that send the data via the network (Data Transmission Layer) to the local server (Data management Layer) to analyze and store them. In addition, a user interface hosted on the local server allows the end-users (Services Layer) to consult the data of their buildings and the graphs of their consumptions in real-time. The service layer is composed of the user interface and the end-users (occupants and managers).
- The Control data: allows the end-users to control the different elements of the building, such as lights, water consumption, etc., via the user interface, which communicates the command to the local server. First, the local server identifies the correct actuator identifier, then broadcasts the command with the actuator identifier in the network. The actuator (Physical Layer) with the same identifier performs the action.

### 2.1. Layer 1: Physical Layer (Buildings)

**Figure 2:**
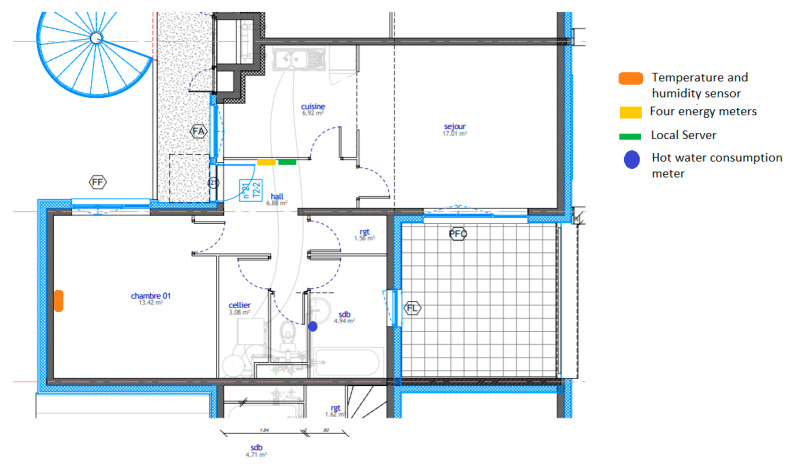
Instrumented building plan.

The physical layer is the first layer of the smart building system. It consists of the buildings, the sensors, and the actuators. Figure 2 shows an instrumented building plan.

**Figure 3:**
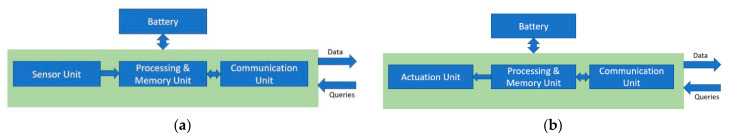
The architecture of (**a**) a wireless sensor (**b**) a wireless actuator.

The physical layer aims to gather data from the environment through wireless sensors and control the building’s equipment using actuators. Figure 3 shows the architecture of this layer. The wireless sensor consists of the sensor unit responsible for capturing the physical quantity and transforming it into a digital value to be processed and stored by the processing and memory unit. When the data is ready, the communication unit sends the data to the local server and waits for requests that the user might send to change the sensor’s parameters, such as the transmission frequency. The wireless actuator consists of control units that execute the queries from the processing to control the building equipment.

#### 2.1.1. Sensors

**Figure 4:**
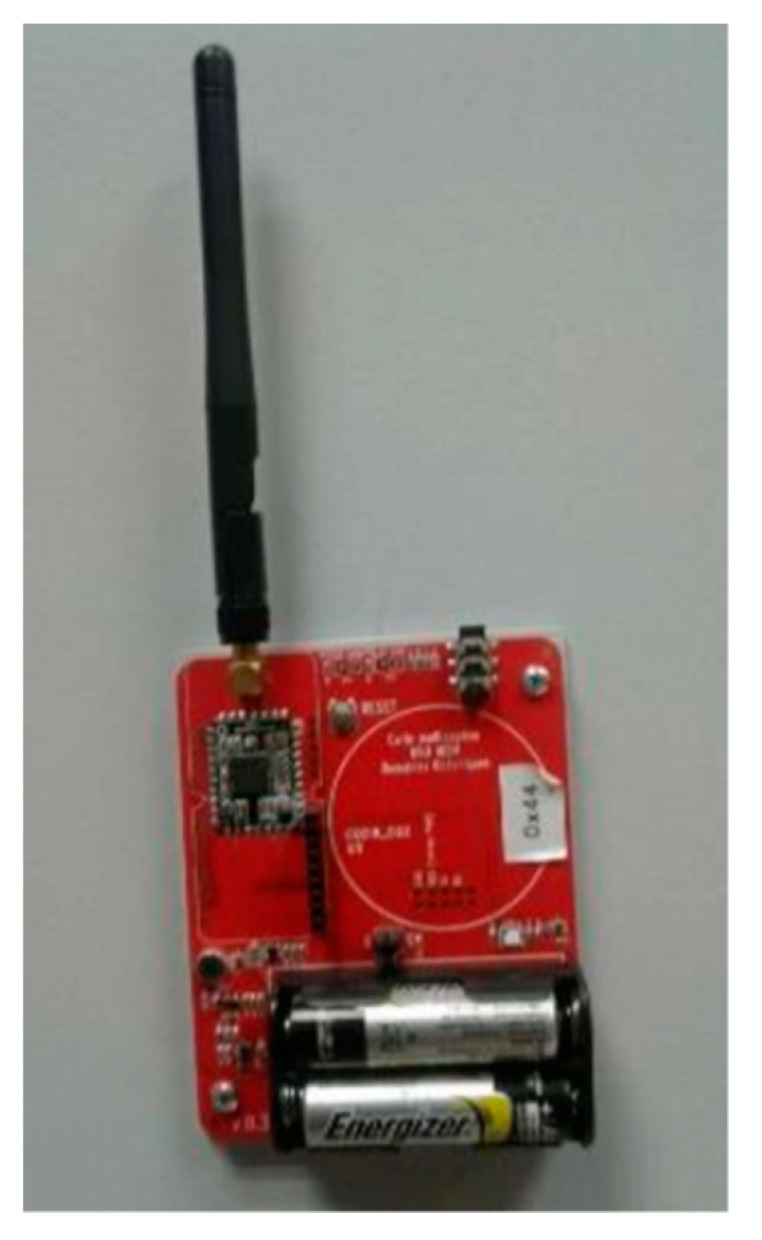
THLN sensor.

- Comfort Sensors:

Comfort sensors are used to quantify occupants’ comfort and indoor air quality. In addition, they allow us to investigate the comfort parameters and their correlation with the other parameters. The system includes the following sensors:

- THLN sensor (Figure 4): This sensor was developed in our laboratory. It contains four sensors: temperature, humidity, lighting, and noise sensors. It is based on the Panstamp NRG module and uses the SWAP protocol to send and receive data from the gateway.
- NODON temperature and humidity sensor [13]: It allows us to monitor both temperature and humidity. It uses Photovoltaic energy. The sensors were calibrated in the factory, but we used trusted sensors for additional verification. In case of a negative verification, sensors were changed or re-calibrated.
- Air quality sensor E4000 [14]: It contains the following sensors: temperature, humidity, CO_2_, and VOC. It uses the EnOcean protocol.
- Eltako multifunction probe FCO2TF65-WG [15]: It is used to measure the CO_2_ concentration, temperature, and humidity.

Table 2 summarizes the technical specifications of the comfort sensors, such as the range, precision, and communication protocol. All the sensors included humidity and temperature sensing to reduce the number of sensors and the cost of the system.

- Consumption Sensors:

Consumption sensors provide information about occupant consumption. The system includes a smart water meter and smart electrical consumption. Two systems were used to measure the electrical consumption:

- Eltako fwz 14-65a [16]: An energy meter sensor with a maximum intensity of 65A and standby loss of only 0.5 Watts. It uses EnOcean protocol to send data to the gateway.
- Panstamp Water Meter: This is a sensor based on the panstamp NRG2 module. We developed it in our laboratory. It sends a packet to the gateway for each litter consumed using the SWAP protocol.

- Security Sensors:

The system includes the following safety and security sensors:

- NODON SDO2105 [17]: The door and window opening detector sends a packet to the gateway every time a person opens or closes a door or a window using the EnOcean protocol.
- GIGACONCEPT DO13-421B-E [18]: The wall-presence sensor sends a packet to the gateway using the EnOcean protocol each time it detects a movement of a person or an object.
- Eltako FRW-WS [19]: It is an EnOcean wireless smoke detector that sends a packet to the gateway when it detects smoke, in addition to producing a loud beep.

#### 2.1.2. Actuators

The following actuators were used to control the building equipment:

- Wireless valve actuator [20]: It is a wireless actuator used to adjust the flow rates to radiators in hot water and steam heating systems. It communicates wirelessly with the local server using the enOcean protocol.
- Wireless actuator light controller FLC61NP-230V [21]: An enOcean wireless light controller with five selectable operating modes.
- Dual-channel wireless switch actuator [22]: It is an enOcean dual-channel wireless switch. Every channel controls a group of 220V electronic lighting loads.

### 2.2. Layer 2: Data Transmission Layer

The data transmission layer ensures the connection between the sensors and actuators and the local server and the connection between the cloud and the local server. Data transmission is based on short-range protocols and long-range protocols.

#### 2.2.1. Short Range Protocols

Short-range protocols are used to communicate between the sensors/actuators and local servers using low-power consumption and low-cost solutions. The following protocols were used:

- **NFC (Near-field communication)** is a standard for contactless radio frequency communication at very short distances (a few centimeters), allowing simple communication between two electronic devices (tag and the reader). Each NFC tag has a unique identifier and can contain a small amount of data.
- **BLE**, also known as Bluetooth Smart, is a short-range communication technology using short-wavelength radio waves with minimal energy. It is designed to enable data collection from sensors that generate data at a very low rate.
- **Z-wave** is a low-power wireless protocol designed for battery or electrically powered devices and is widely used for smart buildings and small-size commercial domains.
- **Wi-Fi**: It is the most used standard for Wireless Local area network (WLAN). It comes with a new standard, IEEE 802.11ah, that provides more scalability, quality of service and energy efficiency.
- **Zigbee** is a short-range technology providing low-power consumption, low complexity, and low-cost advantages. It uses the IEEE802.15.4 standard as its physical layer.
- **EnOcean** is a short-range, low complexity, and secure protocol used by battery-less and wireless sensors.
- **SWAP** is a lightweight, open-source, and low consumption protocol used for short-range communication.

#### 2.2.2. Long-Range Protocols

Long-range protocols are used for communication between local servers and cloud or remote sensors. They include:

- **LTE**: A long-range protocol based on the GSM/UMTS network. It covers fast-traveling devices and provides broadcasting and multicasting services. It is used for high-speed data transfer between mobiles.
- **NB-IoT (Narrow-Band Internet of Things)**: Wide-area cellular connectivity for the Internet of Things provides a low-cost, low-power solution.
- **Lora/LoraWane:** This is a long-range wireless protocol. It is used in long-lived battery-powered devices where energy consumption is of paramount importance. It operates on many ISM bands depending on the region where it is deployed, such as 433 MHz, 868 MHz, or 915 MHz ISM bands.
- **Sigfox**: A French telecommunications operator of the Internet. Sigfox operates in the 868-MHz frequency band. The end device (Sensors) can send up to 140 messages per day, with a payload size of 12 octets.

The proposed system was based on the SWAP and EnOcean protocols, which use energy harvestings such as Piezoelectric and Photovoltaic. The long-range communication was based on the Sigfox network, which is dedicated to IoT. It uses micro-messages (size: 12 bytes) at 10-min time intervals.

### 2.3. Layer 3: Data Management Layer

The data management layer is the core layer of the smart building architecture and the gateway between the sensor/actuator layer and the services layer. It consists of the local server, which performs the following tasks:

- Receive and store the data sent by the sensors;
- Send commands to the actuators;
- Organize the data in a semantic structure of the building;
- Detect sensor errors;
- Perform data visualization;
- Communicate with the central server.

**Figure 5:**
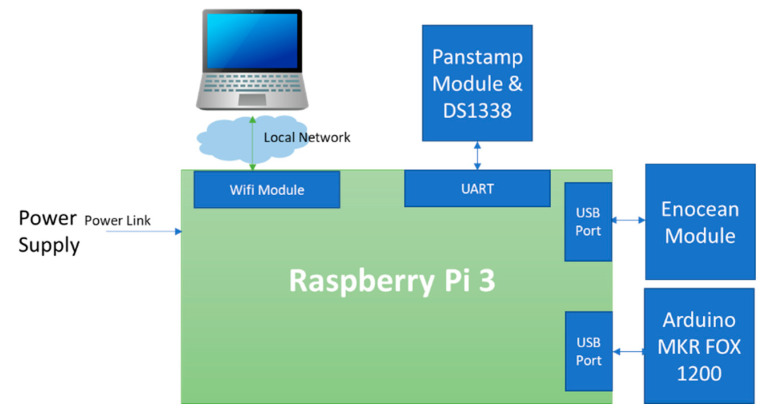
Block diagram of the local server.

Our local server was based on the Raspberry Pi 3 board, a robust, low-cost ARM-based processor board, as shown in Table 3. It performs functions like any computer with the advantage that it has a reduced physical structure [7]. It is equipped with enOcean and Panstamp modules to send commands to actuators and receive data from sensors. It is also connected to the Arduino MKR FOX 1200 board via USB to communicate with the Sigfox network (Figure 5). The Panstamp module contains an integrated DS1338 chip used as a real-time clock module for the gateway.

### 2.4. Layer 4: Services Layer

**Figure 6:**
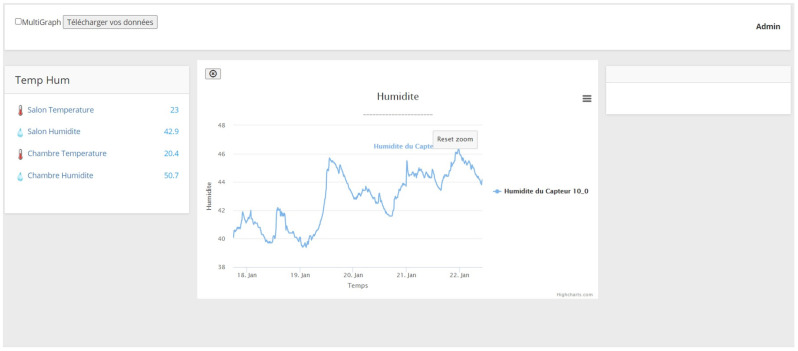
Occupants’ interface.

**Figure 7:**
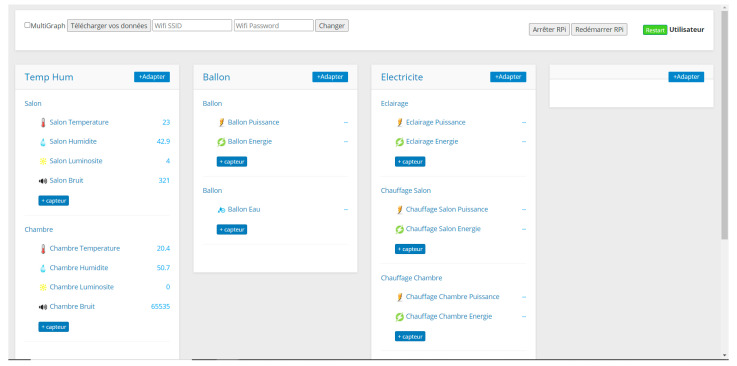
Manager’s interface.

The services layer is the top layer of the smart building architecture. It provides services to users (occupants and managers). It is responsible for data visualization such as the historical and the real-time data of sensors and controlling the building’s equipment. In addition, it manages the interaction between users and the local server. We distinguish two types of users:

- Occupants: They have the right to access real-time and historical data using a graphic interface. They can control the building’s equipment through the interface (Figure 6).
- Managers: They have more privileges and control over the interface (Figure 7), such as:
  - Add, remove, or change sensors/actuators;
  - Add, remove, or change rooms;
  - Activate/deactivate sensors;
  - Export the data to a CSV file;
  - Restart/shutdown the system;
  - Change the sending period of sensors;
  - Check system logs and sensor status.

## 3. Proposed Software Architecture

**Figure 8:**
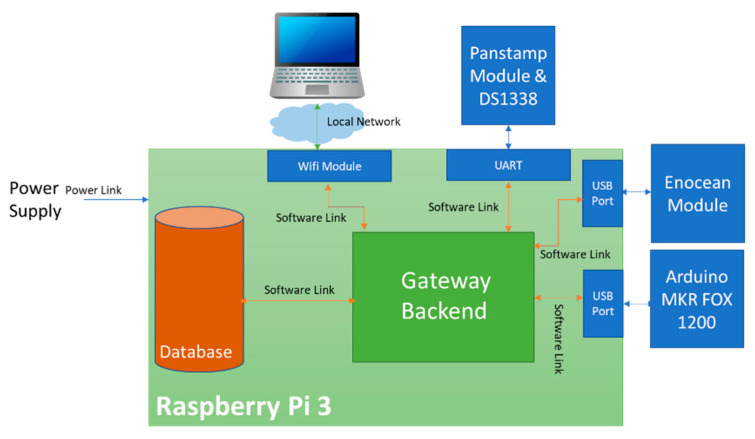
Block diagram of the local server with the software links.

This section presents the software architecture implemented in the local server. It includes two parts, as shown in Figure 8: the database and the software backend architecture.

### 3.1. Database

The database is the main component, which aims to store and retrieve the data collected from sensors. The major types of databases are:

- The relational database is a set of data elements with predefined relationships between them. These items are organized into a set of tables consisting of columns and rows. Tables are used to store information about the objects that should be represented in the database.
- The NoSQL database is an approach to database design that can adapt various data models, including formats with keys, documents, columns, and charts. It is beneficial for working with large, distributed datasets.

**Figure 9:**
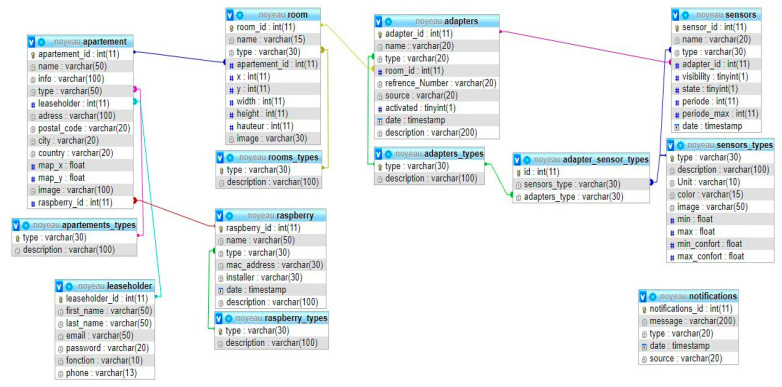
Database Structure.

The system was based on a relational database, which contains several data tables. These tables are connected via a special key to organizing the data in a semantic structure, as shown in Figure 9. This organization allows the installation of our in any building because of its flexibility. We used an annotation adapter, which means a sensor module; for example, the THLN sensor is an adapter containing four different sensors. Each adapter and sensor has its own type. Then, we defined their relationship using table ‘adapter_sensor_types’. This method allowed us to add any sensor or module to our system. When we add a new adapter to our database, the system automatically creates a data table for each sensor of the adapter. This architecture avoids storing all the collected data in one table, which could alter the system performance.

### 3.2. Software Architecture

**Figure 10:**
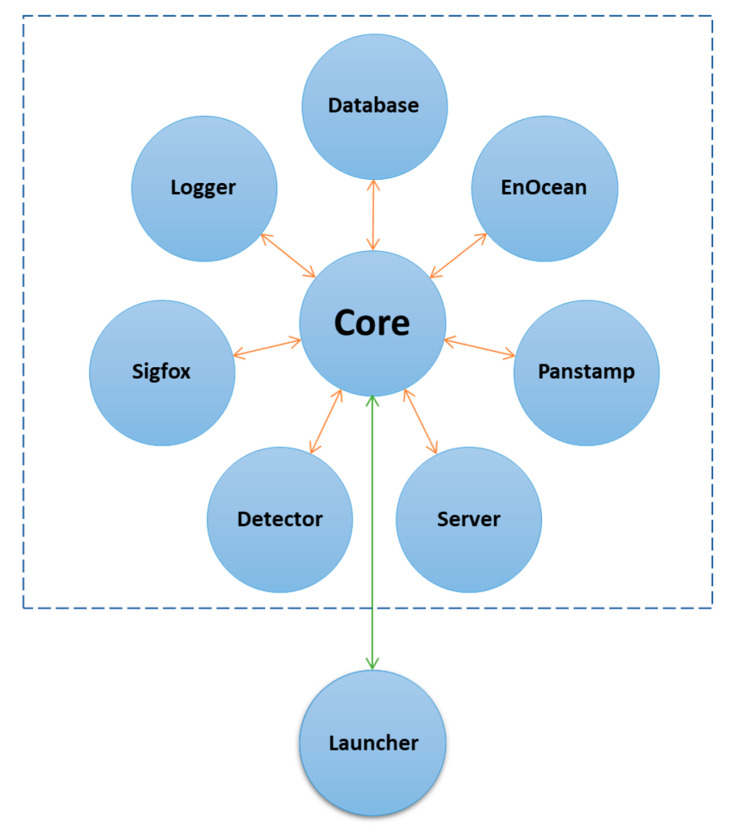
Software modules.

The backend of the local server was based on the open-source server environment nodejs, which uses Javascript. Nodejs was selected for its high performance, scalability, and asynchronous and event-driven programming. The software architecture consists of nine modules, as shown in Figure 10. Each module has a specific role, as described below.

#### 3.2.1. Core

**Figure 11:**
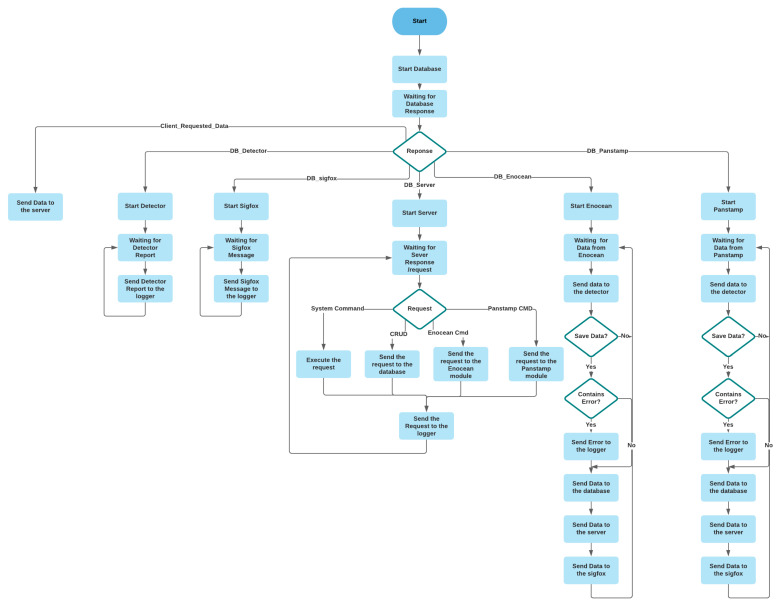
The flowchart of the core module.

The core is the heart of the local server and the maestro that guarantees the communication between the different modules. It also operates the system commands such as restarting, shutting down, and modifying the Wi-Fi parameters of the Raspberry Pi. Figure 11 shows the flowchart of the core.

#### 3.2.2. Launcher

**Figure 12:**
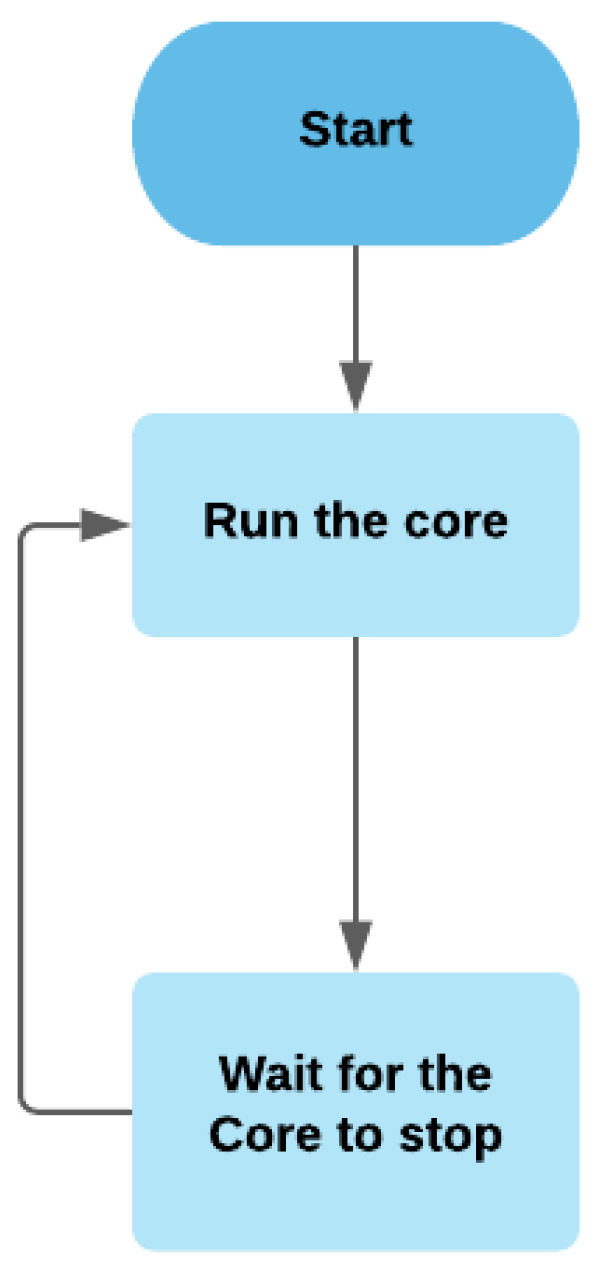
The flowchart of the launcher module.

The launcher is an import module in the software architecture that keeps the core alive whenever it stops, as shown in Figure 12; it is based on a forever-monitor library.

#### 3.2.3. EnOcean

**Figure 13:**
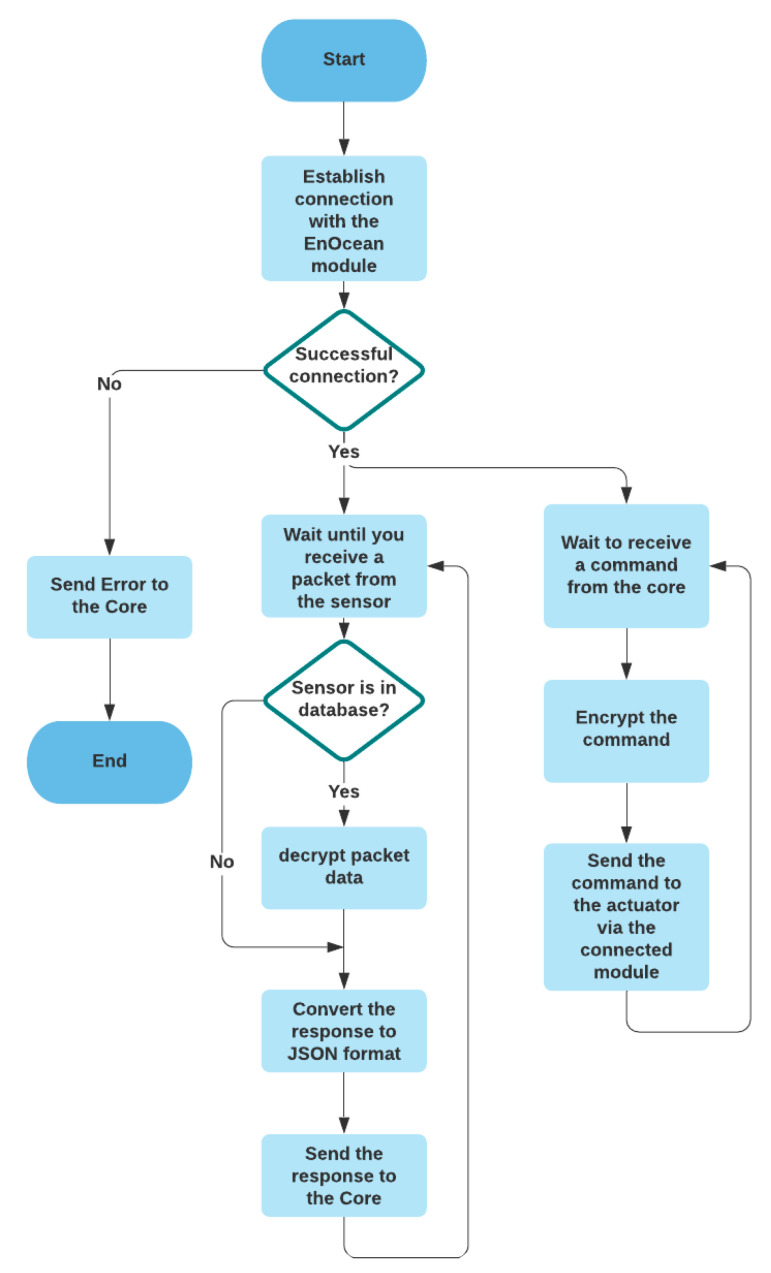
The flowchart the of the EnOcean module.

This module communicates with EnOcean sensors to receive data and send commands and encryption and decryption of packets. Figure 13 describes the mechanism of this module.

#### 3.2.4. Panstamp

This module communicates with Panstamp sensors to receive data, send commands, and encrypt and decrypt the packets. This module works as the EnOcean module.

#### 3.2.5. Detector

**Figure 14:**
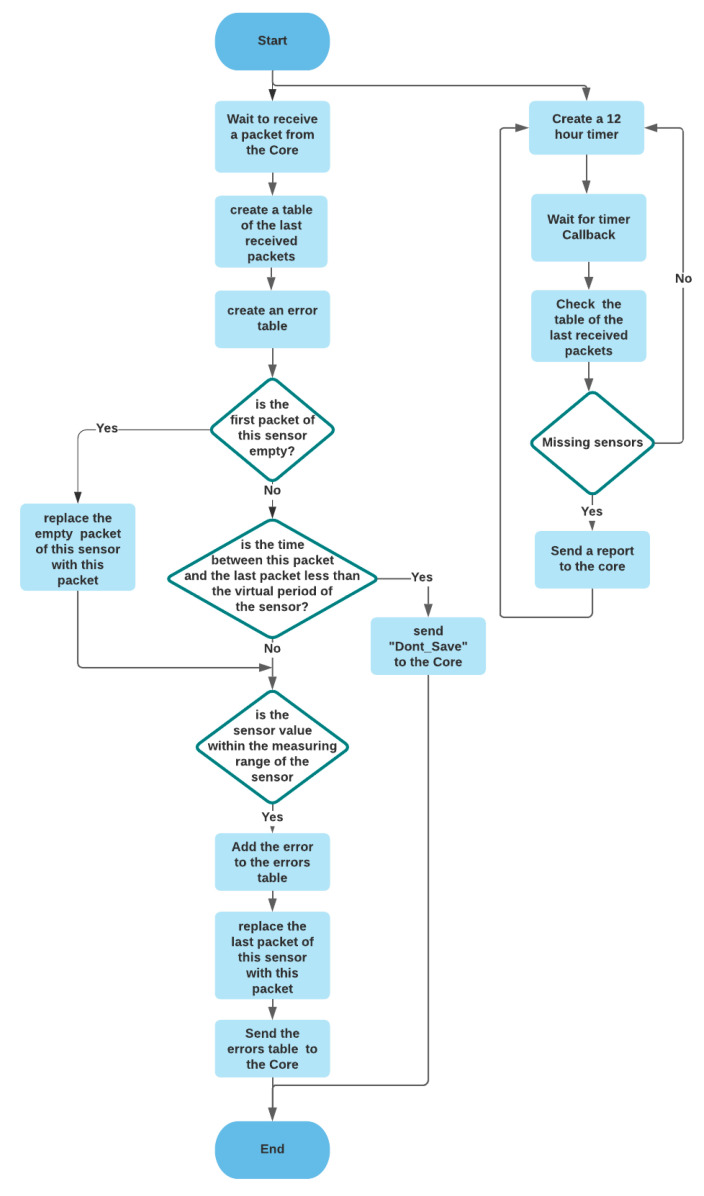
The flowchart the of the detector module.

The detector detects value errors and malfunctions of the sensors by defining the operating rules such as the measuring range of the sensors. In addition, it has a sensor period check function which aims to set a virtual sensor period, for example, if we have a sensor that sends data every five minutes. Still, since we want to store data every 15 min, we defined a virtual period of 15 min for the detector to reject all data sent in less than 15 min. The flowchart of this module is shown in Figure 14.

#### 3.2.6. Logger

**Figure 15:**
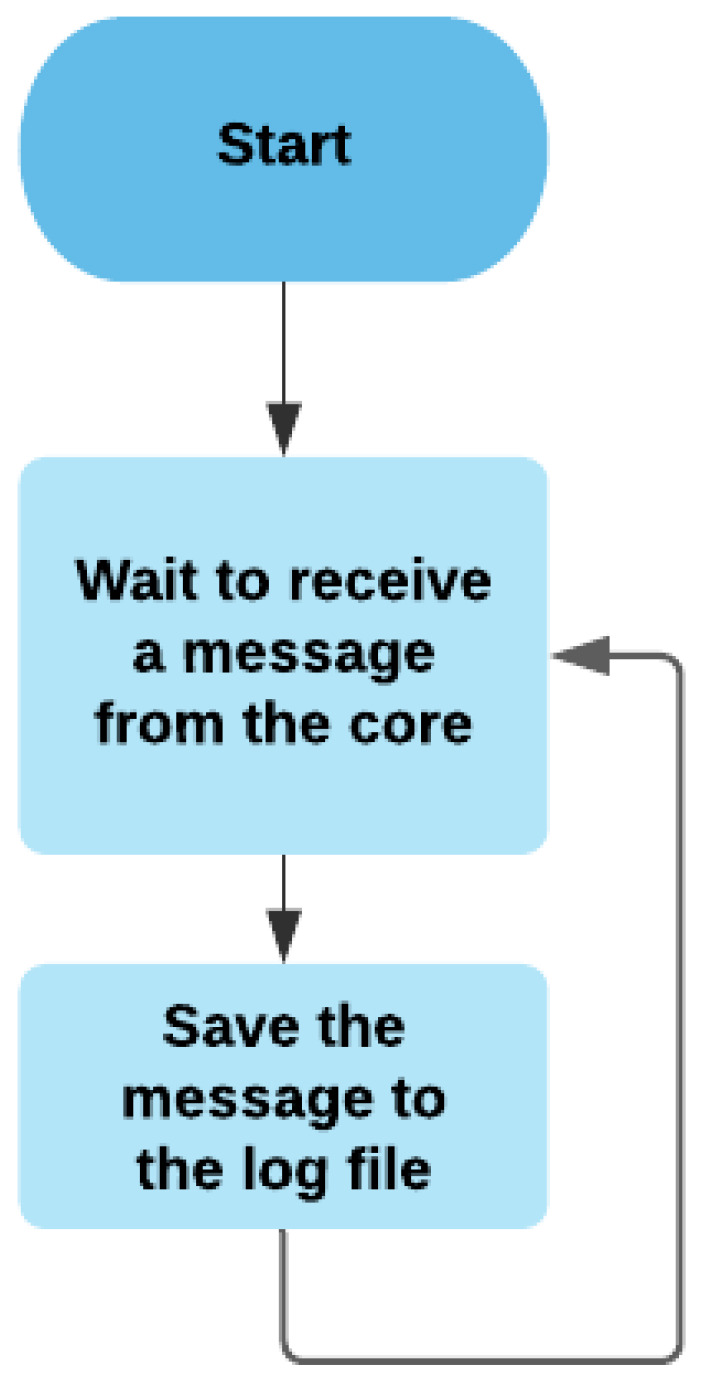
The flowchart of the logger module.

The logger records the important operations operated by the system and the errors sent by the core, as shown in Figure 15.

#### 3.2.7. Web Server

**Figure 16:**
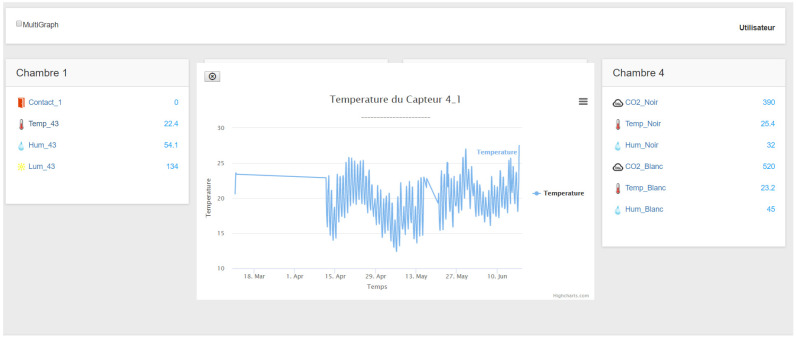
Occupant interface displaying temperature variation.

**Figure 17:**
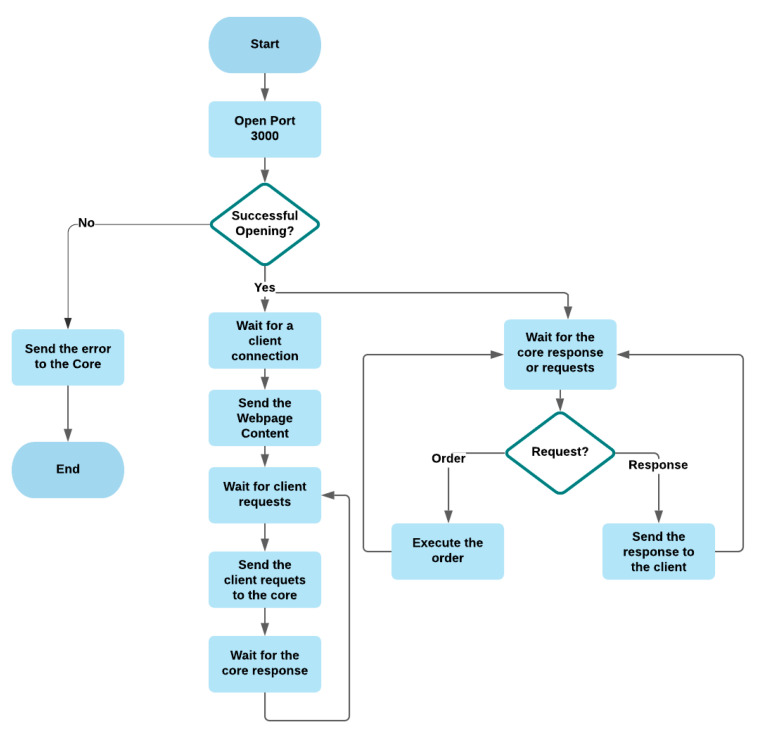
Flowchart of the webserver.

The web server provides a webpage interface (Figure 16) that connects users to the system. It was built with the express library. The web page was developed using HTML, css, and Javascript. The latter was used to perform calculations in the browser to reduce pressure on the Raspberry Pi. In addition, it receives real-time data from the server using the socket-io library. Figure 17 shows the flowchart of the server.

#### 3.2.8. Database

**Figure 18:**
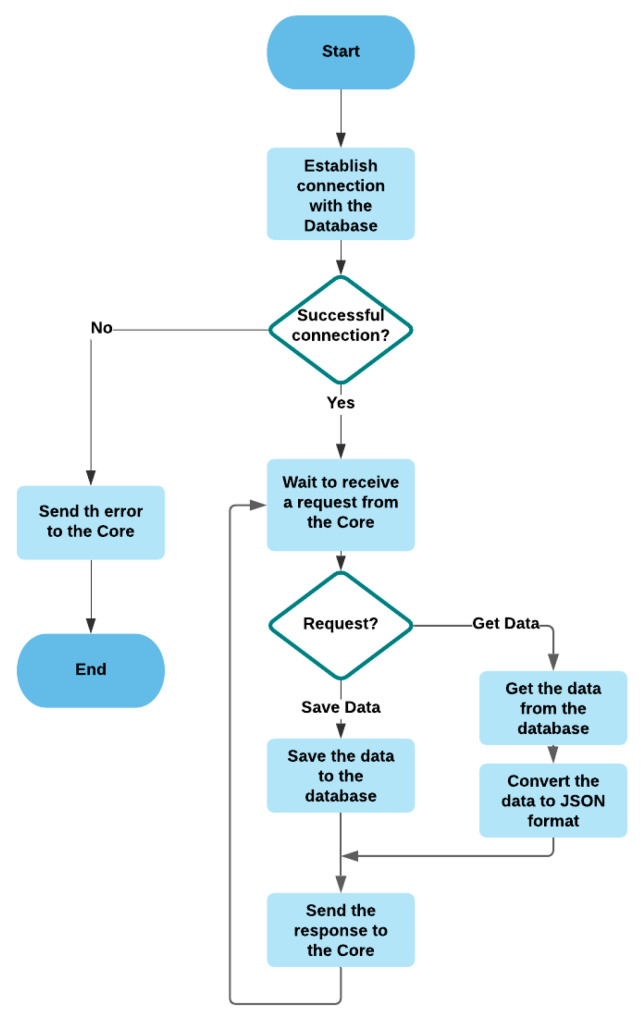
The flowchart of the database module.

The database module is an important module which manages the database (storage and retrieval of data) and creates different copies of the data intended for the other modules to reduce the number of operations with the database, which makes the system faster. Figure 18 shows the flowchart of the database module.

#### 3.2.9. Sigfox

**Figure 19:**
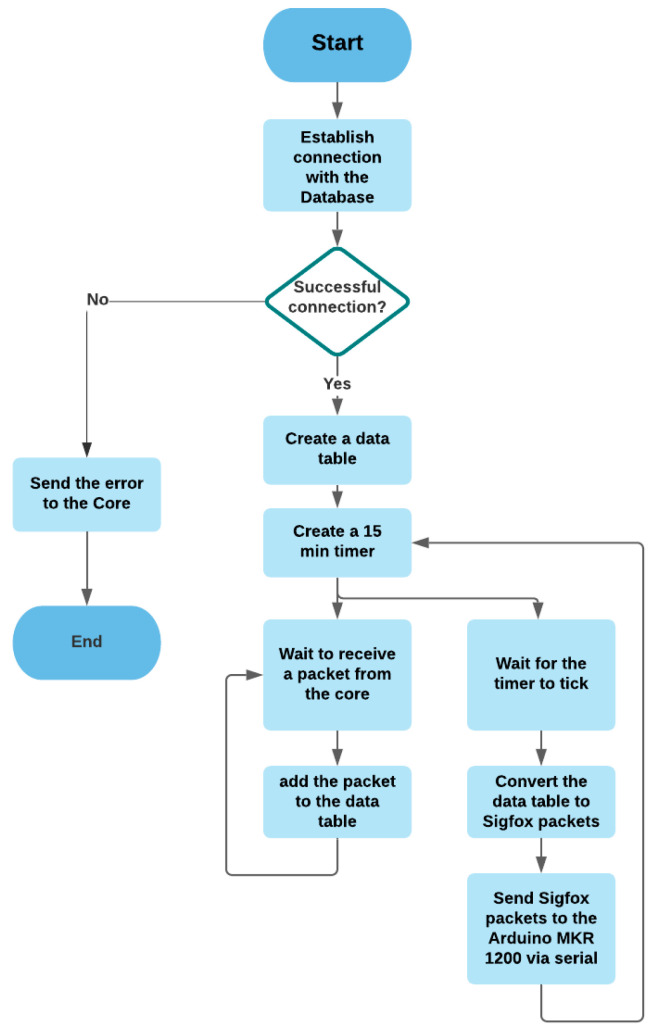
The flowchart of the Sigfox module.

The Sigfox module is responsible for communication with Arduino MKR FOX 1200 to send packets to the cloud. Figure 19 shows the flowchart of the Sigfox module.

### 3.3. Interactions between Software Modules

**Figure 20:**
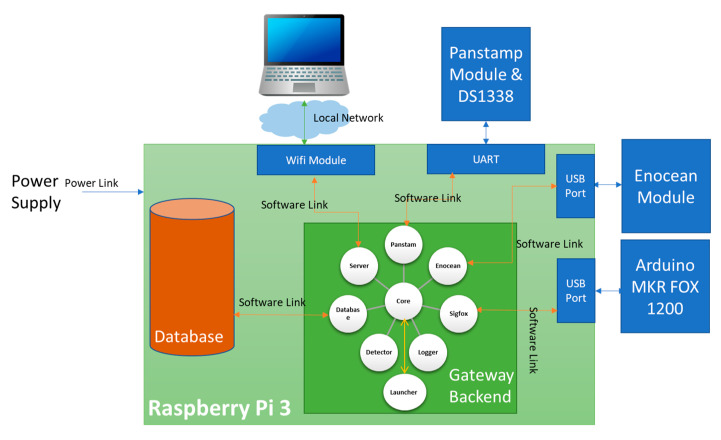
The gateway software and hardware components..

During tests, the software architecture showed flexibility, speed, ability to detect sensor errors, and executes tasks because of the asynchronous processing of nodejs. Figure 20 shows the gateway software and hardware components and their links. The following scenarios were created to break down the interactions of software modules:

#### 3.3.1. System Startup

**Figure 21:**
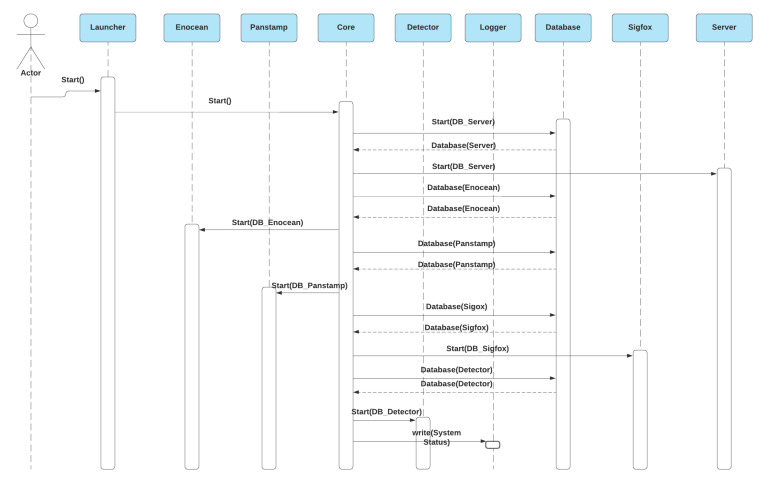
The system startup sequence diagram.

After powering the Raspberry Pi, the system automatically runs the launcher code that starts the system core, forming the database module. The database module creates a custom copy of the database for each module to make the system faster, reduce the number of operations with the database, and sends it back to the core to be distributed to other modules, as shown in Figure 21.

#### 3.3.2. Receipt of Sensor the Packet

**Figure 22:**
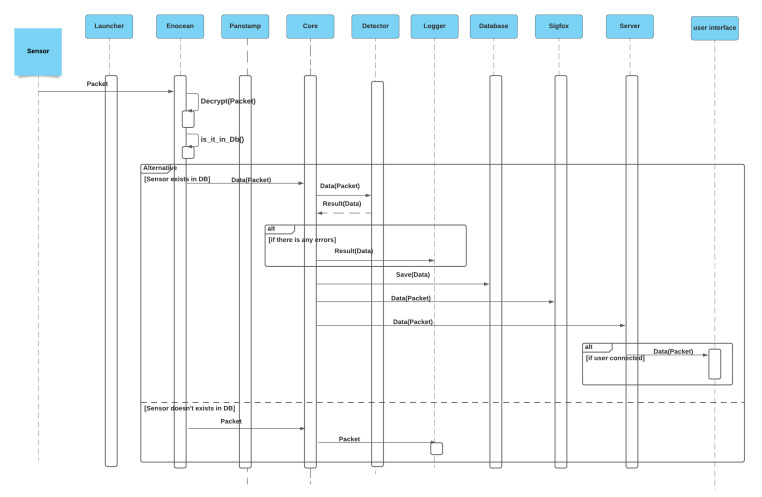
Sensor packet reception sequence diagram.

After receiving the sensor packet via the serial port, the corresponding module checks the existence of the copy of the database. If the copy exists, it sends it directly to the core to be analyzed by the sensor, stored by the database, and sent to the server and Sigfox module. Otherwise, the packet is sent directly to the core to be added to the log file by the logger module. Figure 22 shows the sequence diagram for receiving a sensor packet.

#### 3.3.3. Receipt of Occupants’ Request

**Figure 23:**
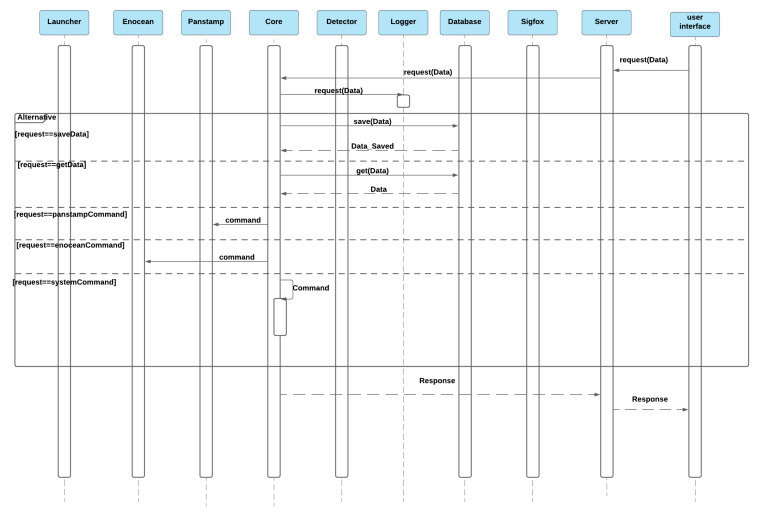
Receipt of occupant’s request sequence diagram.

The system has a web page interface that allows occupants to make requests such as adding sensors, controlling actuators, viewing sensor history, download data, and etc. The occupant’s request goes directly to the server module, which sends it to the core to be executed by different modules if necessary and returns the response via the server module, as shown in Figure 23.

## 4. Case Study and Evaluation

**Figure 24:**
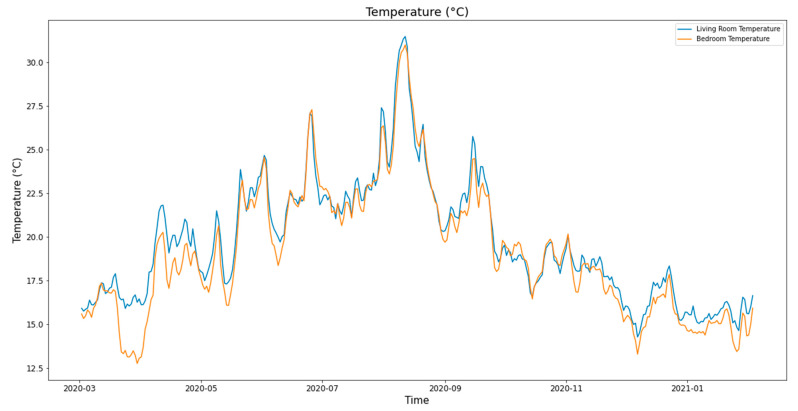
Temperature variation.

**Figure 25:**
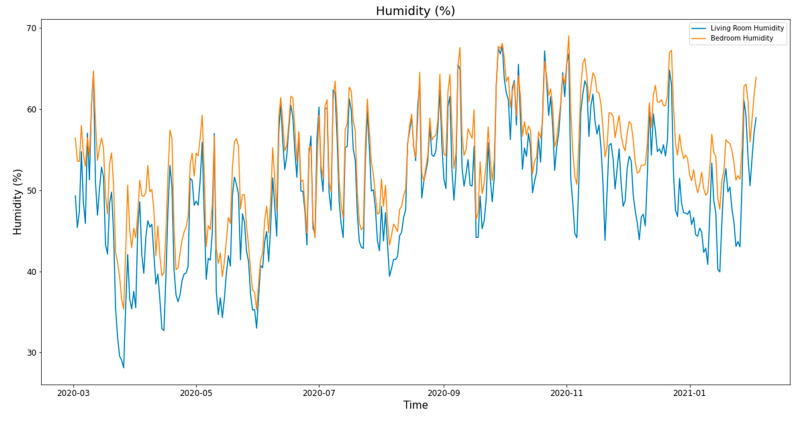
Humidity variation.

To validate the architectural system and its performance, we installed the system in 15 social housing units located in the LMH residence in Wavrin for more than one year between February 2020 and February 2021. In each apartment, we installed the local system with two humidity and temperature sensors, as well as four energy meters and a hot water meter. All the systems worked perfectly. It gathered thousands of data during this period, which allowed us to have interesting data that covers the occupants’ comfort and consumption, which will be presented in other articles. Figure 2 shows the apartment, which was used to validate the proposed system and analyze its performances. Figure 24 and Figure 25 show the variation in the living room and the bedroom of the temperature and humidity, respectively. The temperature in summer increased up to 30 °C, while it decreased in winter down to 12.5 °C. The latter could be related to the heating system shutdown. We observed an important variation in the relative humidity between 35% and 70%.

**Figure 26:**
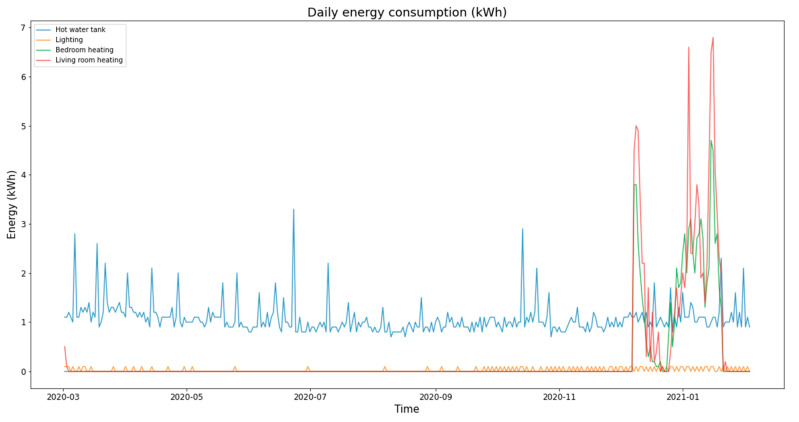
Energy consumption.

Figure 26 shows the variation of energy consumption. It indicates a regular energy consumption for the hot water, negligible energy consumption for the lighting system, and significant energy consumption for the heating system in December and January.

**Figure 27:**
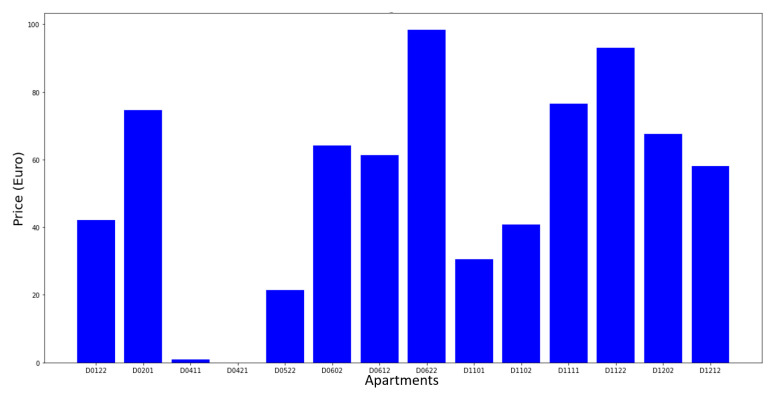
Annual savings in the hot water consumption.

**Figure 28:**
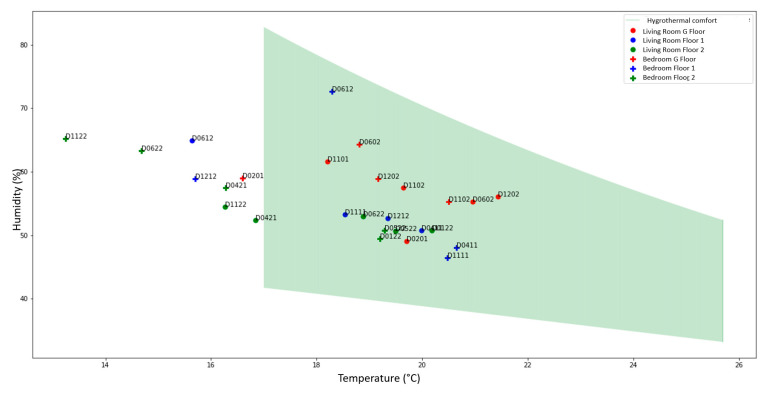
Comfort conditions in the 15 social housing units in winter.

Collected data were analyzed with a focus on the correlation between comfort parameters and the energy consumption. Analysis resulted in recommendations to reduce energy consumption. Figure 27 shows the annual savings for the hot water consumption. It could attain 100 € for some occupants. The hygrothermal comfort was also investigated as illustrated in Figure 28. It showed that the comfort conditions were not respected in some of the apartments because of low temperature.

**Figure 29:**
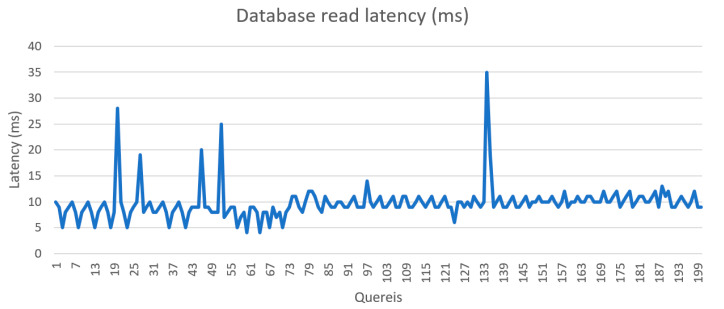
Database read latency.

**Figure 30:**
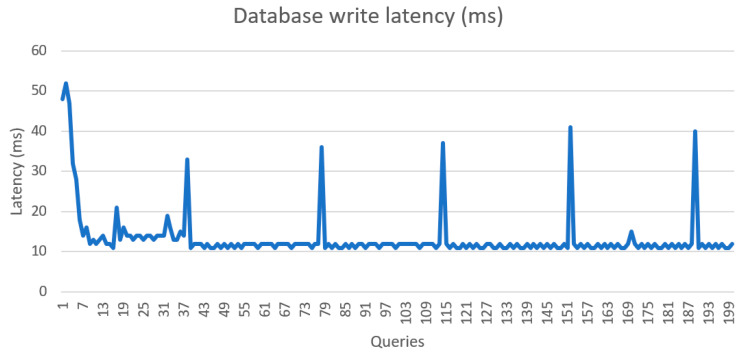
Database write latency.

**Figure 31:**
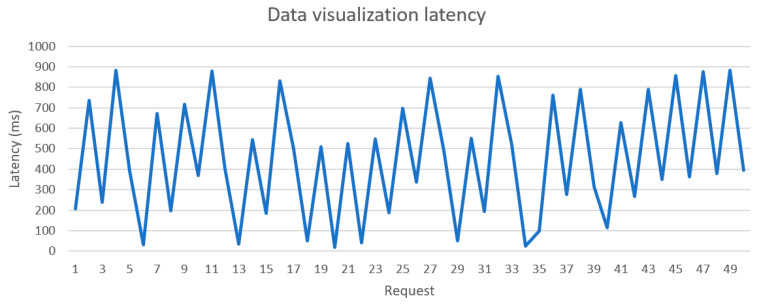
Database visualization latency.

The speed and function of the system was tested and validated during implementation. Figure 29 and Figure 30 show the latency of writing and reading data from the database, respectively. The system took an average of 9.47 ms to record sensors records and 13.4 ms to read data from the database. Figure 31 shows the latency of data transmission from sensors to the local units was around 447 ms.

## 5. Conclusions

This paper presented the design and construction of a smart building system that enables both buildings’ monitoring and control. This system is characterized by its simplicity, reliability, low cost, and ease of construction and installation. In addition, the system could be extended easily to host new components and services.

The system provides a large set of smart services through monitoring indoor comfort, air quality, fluid consumption, and building safety. It also allows automatic or online control of the building equipment. The system integrates a friendly environment with a graphic interface.

The paper’s novelty concerns the design and construction of a modular smart monitoring system, which integrated nine interactive modules that ensure high performances in terms of low energy consumption, real-time monitoring, error detection, and scalability.

The paper presented a detailed description of the hardware and software components and the specifications of the materials and the technologies.

The system was used in monitoring fifteen social housing units. Collected data were used for the verification of the indoor comfort as well as energy and hot water consumption. Data analysis showed abnormal energy consumption in some units.

Academics and professionals could use this information to extend the capacity of the existing smart building system.

In future work, the collected data will be used to analyze the comfort conditions, energy, and water consumption in residential buildings. Correlation analyses will also be conducted to investigate the relationships between comfort conditions and energy consumption. In addition, data will be used to analyze the impact of the occupant profile on energy and water consumption.

## Figures and Tables

**Table 1:** Comparison of the proposed solution with other systems from the literature.

Solution Reference	[2]	[3]	[4]	[5]	[7]	[8]	[9]	Proposed System
Webpage, mobile application	Yes	No	Yes	No	Yes	Yes	Yes	Yes
Support different types of sensors	No	Yes	No	No	Yes	Yes	Yes	Yes
Support existing sensors on the market	No	No	No	No	No	No	No	Yes
Multi-factor authentication	Yes	No	No	Yes	No	No	No	Yes
Communication with the cloud	No	Yes	Yes	Yes	Yes	Yes	No	Yes
Support different occupants’ profiles	No	No	No	No	No	No	No	Yes
Detection of sensor errors	No	No	No	No	No	No	No	Yes
Configuration of the sensors via the webpage	No	No	No	No	No	No	No	Yes
System Logger	No	No	No	No	No	No	No	Yes
Contains actuators	Yes	No	Yes	yes	Yes	Yes	Yes	Yes
Export and download data (csv, Excel)	No	No	No	No	No	No	No	Yes
visualization of charts	No	Yes	Yes	No	No	Yes	Yes	Yes
Database	Firebase	Mysql	DynamoDB	Firebase	MongoDBMysql	--	--	Mysql
Gateway/Local server	--	Raspberry	Intel Edison	--	--	--	--	Raspberry Pi
Wireless	Wi-Fi	Wi-Fi	Wi-Fi	Wi-Fi	Bluetooth, Wi-Fi	--	Z-Wave	Wi-Fi, EnOcean, SWAP, Sigfox
Chipset	Wemos D1 Mini V3.0.0	NodeMCU	--	--	Arduino Uno/Pro Mini, HC 05, ESP8622	Arduino	Z-Wave chipset	EnOcean sensors, ESP32, Atmega328p
Support Data Analysis	No	Yes	No	No	No	Yes	No	Yes
Manage system crashes	No	No	No	No	No	No	No	Yes
Notification	No	Yes	yes	Yes	No	No	No	Yes
Is the architecture of the database described?	No	No	No	No	No	No	No	Yes
Are the software components described?	No	No	No	No	No	No	No	Yes

**Table 2:** Technical specifications of the comfort sensors.

Name	Sensor	Range	Precision	Protocol
THLN Sensor	Temperature	−10 to 85 °C	±0.4 °C	SWAP
	Humidity	0 to 100%	±3%	
	Lighting	0 to 500 Lux	±10 Lux	
	Noise	20 to 90 dB	±2 dB	
NODEON temperature and Humidity sensor	Temperature	0 to 40 °C	±0.16 °C	EnOcean
	Humidity	0 to 100%	±3%	
E4000 Probe	Temperature	0 to 50 °C	±0.3 °C	EnOcean
	Humidity	10 to 90%	±3%	
	CO_2_	390 to 3500 ppm	±100 ppm	
	VOC	Max 300 ppm	±0.01 ppm	
FCO2TF65-WG	Temperature	0 to 51 °C	±0.5 °C	EnOcean
	Humidity	0 to 100%	±0.5%	
	CO_2_	Max 2550 ppm	±10 ppm	

**Table 3:** Raspberry Pi 3 technical specifications.

CPU	4 × ARM Cortex-A53, 1.2GHz
GPU	Broadcom Video core IV
SoC	Broadcom BCM2837
RAM	1GB LPDDR2 (900 MHz)
Storage	microSD (16Go in our case)
Networking	10/100 Ethernet,2.4 GHz 802.11n wireless, Bluetooth 4.1 Classic and BLE
GPIO	40-pin header
Ports	HDMI, 3.5 mm analog audio-video jack, 4 × USB 2.0, Ethernet, Camera Serial Interface (CSI), Display Serial Interface (DSI)

## Data Availability

Not applicable.
